# Biomechanical Evaluation of Flexor Tendon Repair: Double Loop Technique with Epitendinous Suture

**DOI:** 10.5704/MOJ.2507.001

**Published:** 2025-07

**Authors:** AN Sadagatullah, S Raghu, M Paiman, S Ismail, MH Jusoh

**Affiliations:** 1 Department of Orthopaedics, Universiti Sains Malaysia, Kubang Kerian, Malaysia; 2 Department of Anatomy and Physiology, Universiti Sultan Zainal Abidin, Kuala Terenggganu, Malaysia

**Keywords:** biomechanics, flexor tendon, monofilament polypropylene suture, repair

## Abstract

**Introduction:**

There are various methods used to repair lacerated tendons. The minimum requirement for the best results and lowest rupture rate is the four-strand repair technique. The cruciate type of repair is among the most popular methods available but is very technical and requires expertise. An easier two-double-loop method for tendon healing is suggested in this study. This study assessed the biomechanical properties of two well-known tendon repair techniques—the modified Kessler and cruciate approaches—and two lesser-known double-loop techniques for tensile strength, stiffness, and failure mode.

**Materials and Methods:**

Twenty-four adult chickens' Achilles tendons were randomly divided into three groups and sutured with a four-strand core suture using the four-strand modified Kessler technique, the four-strand cruciate technique, and the two-double-loop approach. Twenty-four more adult chicken Achilles tendons were acquired, and they were randomly assigned to the same three groups along with an extra running epitendinous repair. A synthetic, non-absorbable monofilament polypropylene suture was used for all repairs.

**Results:**

The four-strand modified Kessler, and the four-strand cruciate procedures had the lowest mean ultimate tensile strength, whereas the two double-loop techniques had the strongest. The results were dramatically impacted by using an epitendinous suture during test analysis.

**Conclusion:**

The strongest and comparatively less technically complex technique used in this investigation was the two-double-loop, four-strand core suture method. The significance of the extra strength that the epitendinous suture gave was clear. Using this in a clinical setting is recommended for hand flexor tendon injuries.

## Introduction

With the overarching shift in society towards service-based and do-it-yourself work, this phenomenon has escalated the number of hands injuries^1^. On average, hand and wrist injuries are among the most common injuries across ages that pose the risk of disability^2^. The National Electronic Injury Surveillance System reported that hands and fingers are the most common anatomical sites exposed for lacerations and fractures in the USA. In 1996, approximately 1 million workers were reported to have hand injuries, according to Morbidity, Mortality Weekly Report, 1998. The hand flexor tendon is susceptible to lacerations due to its outstanding anatomy. This tendon lies close to the skin, making it susceptible to lacerations commonly occurring in daily life activities in professions and sports. Tendon healing upon injury depends on age, overall health condition, scar formation disposition, motivation, injury risk based on Verdan’s zones, injury type, synovial containment, and surgical techniques.

Surgery is the only recourse in managing flexor tendon injury. Both ends need to be tied up together by an unleashing knot. The healing process involves intrinsic and extrinsic mechanisms, originating from the migratory reaction of endotenon and epitenon towards the wound site or outside inflammatory and fibroblast cells such as synovium or tendon sheath towards the wound sites^3^. The surgical procedure is challenging since the tendon must be glided as early as day four post-operation to improve tendon healing besides reducing adhesion formation. This early mobilisation requires rehabilitation from a structured protocol, making it challenging since it may pose the risk of rupture^4^.

For instance, current techniques, such as the modified Kessler and cruciate techniques, have long been used for repairing hand flexor tendon injuries. It is a widely used technique by hand surgeons for its effectiveness^5^. However, the concern was raised due to the possibility of forming a gap between the tendon ends, which may lead to impaired tendon healing and decreased functional outcomes. On the other hand, it does not provide sufficient strength to withstand the forces placed on the repaired tendon during early mobilisation. It inflicts rupture in cases of high-demand activities^6^. Furthermore, the knots used in the modified Kessler suture technique can sometimes irritate joint capsule and soft tissue, which leads to adhesion that restricts finger motility, discomfort in some patients, or pain and even infection^7^. On the other hand, the cruciate technique commonly employed involves placing sutures in a cross-shaped pattern. However, this technique requires twice the force to generate a small gap, potentially leading to suboptimal healing and impaired function^8^. Bad clinical results and increased adhesion formation have been associated with gaps larger than 2mm^9-11^. Additionally, Silfverskiöld *et al*^12^, found that at year 1, gap formation and interphalangeal motion had an inverse association. They raised concerns about insufficient strength provided by the cruciate technique and the risk of adhesion, which limits tendon gliding, thus decreasing finger joint mobility and inevitably impairing hand functionality.

Hence, this study was conducted to evaluate these techniques. The double-loop technique was proposed, and its biomechanical evaluation has been performed and compared to the modified Kessler and cruciate techniques. Eventually, this study will also determine the epitendinous suture effects of these approaches on the flexor tendon repair.

## Materials and Methods

A 3/8 circular reverse cutting needle made of synthetic, non-absorbable monofilament polypropylene suture was utilised (size 4-0 for the core suture and 6-0 for the epitendinous suture). Three methods, both with and without epitendinous sutures, were evaluated. The Achilles tendons of chickens were selected as our sample for their availability and size matching with the human flexor tendon^13^. To determine its sample size, PASS version 14 software was used. With eight samples per technique and 24 samples for each type of independent variable (epitendinous and non-epitendinous), the power was set at 0.90, and the critical value alpha was set at 0.05, resulting in a total sample size of 48.

**Fig. 1:**
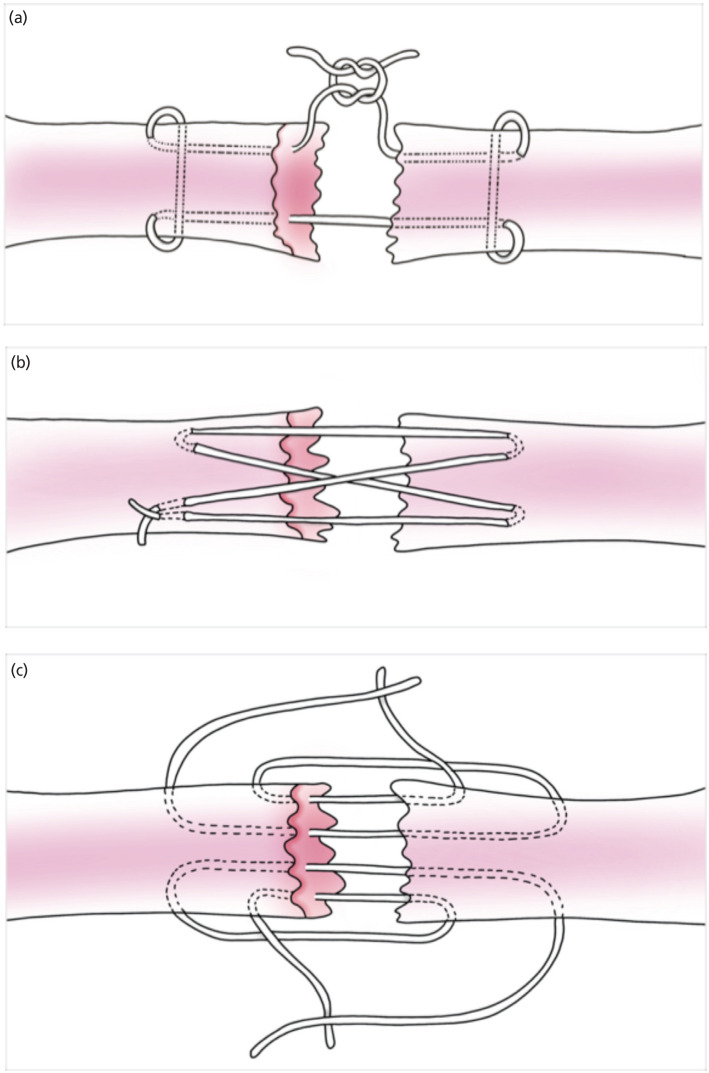
(a) Modified Kessler – Diagram simplified to show a two-core technique repeated to obtain four cores, (b): cruciate technique and (c): two double-loop technique.

We randomly selected 48 Achilles tendons from 24 adult chickens that had just been killed for commercial purposes. Every tendon was wrapped in gauze that had been soaked in saline. There were six groups of eight tendons in all. The same surgeon performed all the repairs, transversely cutting each tendon at the midpoint with a sharp surgical blade and suturing them immediately. All tendons underwent similar repair procedures, with each group receiving an allocated core suture placement followed by the placement of peripheral sutures. Every peripheral suture was standardised using the same knot type and an identical purchase of 3mm from the lacerated region with six continuous needle passes. Three distinct four-strand core suture techniques were employed: the modified Kessler (Fig. 1a), the cruciate (Fig.1b), and the two double- loop approaches (Fig. 1c). Ten millimetres from the tendons' lacerated ends, the core repairs' sutures grabbed them. The Vernier calliper was used to locate the suture biting point. The same knot was used to secure each repair.

**Fig. 2:**
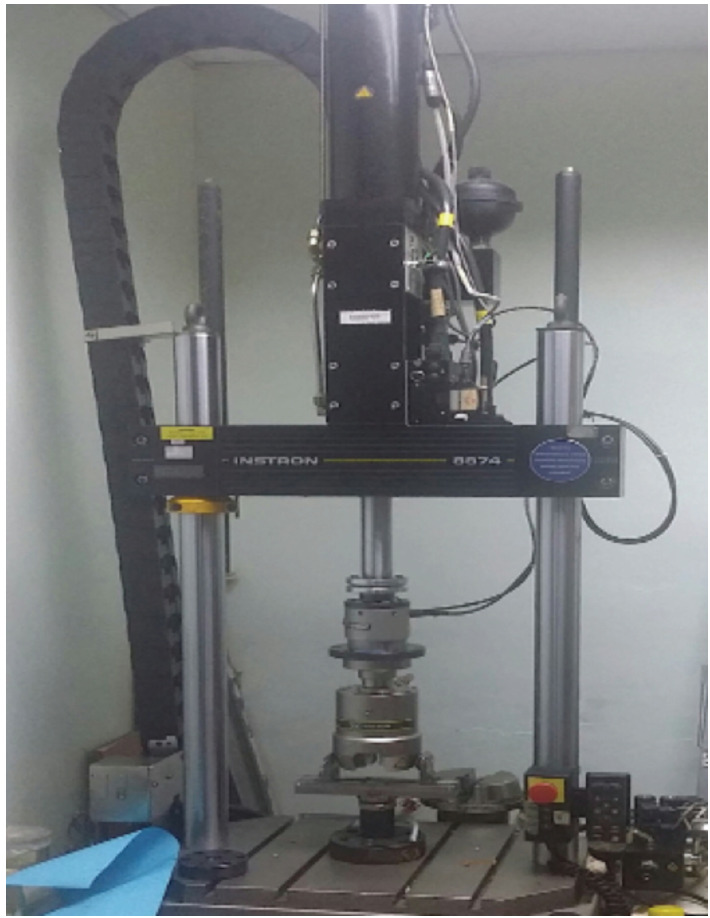
8874 Biaxial Servohydraulic Fatigue Testing, INSTRON [Illinois, USA] from Dental Laboratory, Universiti Sains Malaysia.

For mechanical testing evaluation, 8874 Biaxial Servohydraulic Fatigue Testing, INSTRON [Illinois, USA] from Dental Laboratory, Universiti Sains Malaysia (Fig. 2), was used to measure the ultimate tensile strength in the sutured tendons. Every tendon was constricted at both ends, 15mm from the repair site. At a loading rate of 10mm per minute, the tensile force was applied to the connecting axis. Millimetres were used to compute the load-to-failure curves. Measurements were made on the stiffness and ultimate load of failure. A stiffness range of 5 N to 70 N was measured. The ultimate load to failure, mode of failure, and stiffness of every tendon were examined and tested. Failure was described as suture breakage, knot failure, or core suture pull-out (intact sutures slipping out of a tendon) (suture rupture).

To emphasise the significance of an epitendinous repair, a multivariate statistical analysis of the Wilks' Lambda test was carried out between the three procedures and their kinds (non-epitendinous and epitendinous). Lastly, with an alpha value set at p<0.05, a Fischer Exact test was run to assess the significance of suture failure with respect to the kind of suture.

## Results

The three procedures and types of sutures showed significant differences (p<0.05) in the multivariate tests conducted using Wilks' Lambda test (Table I). (epitendinous and non-epitendinous). Tensile strength measurements showed that the tendons with an epitendinous repair had greater superior tensile strength. The average result for the cruciate non-epitendinous group was 19.6 N, while the epitendinous group had a substantially higher reading of 45.6 N. The modified Kessler that is non-epitendinous recorded a value of 22.4 N. The epitendinous group, however, measured an average of 41.4 N. The non-epitendinous group recorded 30.3 N with the two double-loop procedures, whereas the epitendinous group recorded 45.7 N.

**Table I:** Multivariate tests analysis between three techniques and two suture types. Multivariate Tests^a^

Effect		Value	F	Hypothesis df	Error df	Sig.
Technique	Wilks' Lambda	.388	8.069^b^	6.000	80.000	.000
Type	Wilks' Lambda	.412	19.042^c^	3.000	40.000	.000

Notes – ^a^ Design: Technique + Type, ^b^ Exact statistic, ^c^ The statistic is an upper bound on F that yields a lower bound on the significance level.

Tensile strength did not significantly differ between the well-established cruciate and modified Kessler methods. Nonetheless, the recently suggested approach—the two double-loop technique—was noteworthy. The epitendinous suture exhibits a high tensile strength as well. The primary cause of failure in the epitendinous sutures was knot breaking. Conversely, the majority of non-epitendinous type suture failures were attributed to suture pull-out, which was statistically significant at p=0.015 (Table II).

**Table II:** Analysis of suture failure from different types of suturing technique using Chi-Square.

	Value	Df	Asymp. Sig. (2-sided)	Exact Sig. (2-sided)	Exact Sig. (1-sided)
Pearson Chi-Square	7.378a	1	.007		
Continuity Correctionb	5.829	1	.016		
Likelihood Ratio	7.668	1	.006		
Fisher's Exact Test				.015	.007
Number of Valid Cases	48				

Notes: ^a^ Zero cells (0.0%) have expected count less than five. The minimum expected count is 8.50, ^b^ Computed only for a 2x2 table.

Four of the twenty-four samples (non-epitendinous group) with only a core suture failed because of knot breakdown. These four mishaps were all from the group using the modified Kessler approach. This suggests that the highly skilled suturing approach degraded the quality of the knot tying.

All epitendinous suturing failure started at the peripheral site while enduring the tensile load, and then the breakage spread to the knot. The core sutures failed at a higher tensile load. This agrees with other researchers, who also reported the importance of an added epitendinous suture (Table III). The significant differences between the two types of sutures (epitendinous and non-epitendinous) that failed due to maximum stress, strain, and force (p<0.05) and the three techniques that resulted in suture failure due to maximum strain (p=0.039) and maximum stress (p=0.024) are displayed in this table. However, there was no statistically significant difference in force between the three approaches.

**Table III:** Multivariate Test Analysis for independent techniques and suture types with their combined effects on maximum strain, stress and force causing suture failure.

Source	Dependent Variable	Type III Sum of Squares	Df	Mean Square	F	Sig.
Technique	Maximum Strain	97.14	2	48.57	3.520	.039*
	Maximum Force	359.51	2	179.76	2.162	.128
	Maximum Stress	14.53	2	7.27	4.099	.024*
Type	Maximum Strain	249.22	1	249.22	18.062	.000*
	Maximum Force	4864.58	1	4864.58	58.512	.000*
	Maximum Stress	101.39	1	101.39	57.200	.000*

Notes- ^a^ R squared = .974 (Adjusted R squared = .970), ^b^ R squared = .946 (Adjusted R squared = .939), ^c^ R squared = .937 (Adjusted R squared = .928)

A post-hoc Tukey test was used to establish multiple comparisons between the three distinct suture procedures with respect to strain, force, and stress. Table 4 demonstrated that the modified Kessler and cruciate approaches had an average tensile strength of 32 N and 38 N, respectively. In contrast, the four-strand two double-loop technique had the maximum tensile strength. The two double-loop and cruciate approaches differed significantly from one another at the maximum strain that led to suturing failure (p=0.047), and the two double-loop and Kessler techniques differed significantly from one another at the maximum stress that led to suture failure (p=0.018). No statistically significant difference was seen in the analysis conducted between the four-strand modified Kessler technique and the widely recognised four-strand cruciate approach.

**Table IV:** Multivariate analysis for comparison of maximum strain, maximum force and maximum stress between cruciate, Kessler and two-double-loop technique by post-hoc (Tukey) analysis.

Dependent Variable	(I) Tech	(J) Tech	Mean Difference (I-J)	Std. Error	Sig.	95% Confidence Lower Bound	Interval Upper Bound
Strain	Cruciate	Kessler	.47	1.31	.931	-2.71	3.66
		Two double-loop	3.22*	1.31	.047	.036	6.42
	Kessler	Cruciate	-.47	1.3	.931	-3.66	2.72
		Two double-loop	2.75	1.31	.103	-.44	5.94
	Two	Cruciate	-3.2*	1.31	.047	-6.42	-.036
	double-loop	Kessler	-2.75	1.31	.103	-5.94	.44
Force	Cruciate	Kessler	.67	3.22	.976	-7.16	8.50
		Two double-loop	-5.44	3.22	.222	-13.27	2.39
	Kessler	Cruciate	-.67	3.22	.976	-8.50	7.16
		Two double-loop	-6.11	3.22	.152	-13.94	1.72
	Two	Cruciate	5.44	3.22	.222	-2.39	13.27
	double-loop	Kessler	6.11	3.22	.152	-1.72	13.94
Stress	Cruciate	Kessler	.63	.47	.381	-.51	1.77
		Two double-loop	-.72	.47	.292	-1.86	.43
	Kessler	Cruciate	-.63	.47	.381	-1.77	.51
		Two double-loop	-1.35*	.47	.018	-2.49	-.20
	Two	Cruciate	.72	.47	.292	-.42	1.86
	double-loop	Kessler	1.35*	.47	.018	.20	2.49

Based on observed means. The error term is mean square (error) = 1.773. *The mean difference is significant at the .05 level.

## Discussion

Three suturing procedures using the widely used synthetic, non-absorbable monofilament polypropylene suture material were compared in this study (prolene). The four-strand cruciate technique and the four-strand two double-loop technique were compared to the well-established four-strand modified Kessler suture technique's tensile strength. To assess their increased mechanical strength and gapping resistance, epitendinous sutures were used in both the experimental and control groups^14^. The Achilles tendon of chickens was used as our sample for its similarity in size to the human flexor tendon, besides demonstrating improved mechanical strength and matrix deposition compared with the cell-free scaffold.

Our study shows that the two double-loop techniques are biomechanically the strongest and possess the highest tensile force upon repair compared with the established method of cruciate and modified Kessler techniques. The two double-loop techniques were technically less demanding compared to the modified Kessler and cruciate techniques^15^. There were 12 suture bites in a modified Kessler four-strand technique compared to the eight suture bites required for both the cruciate and two double-loop techniques. Despite having an equal number of suture bites, the two double-loop techniques were easier to perform, less time-consuming and less traumatic when handling tendon edges.

The outcome of tendon repair depends on several factors. A robust and durable suture is crucial for tendon repair. However, since the tendon must run through confined sheaths and pulleys, a bulky repair must be avoided to allow early mobilisation. The primary purpose of the tendon is to efficiently transfer force to the bone. Therefore, it must be strong and viscoelastic enough to hold mechanical energy and bear stress during range-of-motion exercises^16^. A tendon repair with enough tensile strength will allow for early mobilisation^17^, which will stop adhesions from forming^18^, encourage tendon healing^19^, and improve the prognosis^20^ in the clinical setting.

A trauma-free surgical approach with a tendon anchorage of at least 10mm from the traumatic incision is frequently cited as the optimal flexor tendon restoration^21,22^. The suture should be simple to work with, maintain a tight knot^23,24^, and not interfere with the tendon's vascularity or its ability to glide through its sheath and pulleys^25,26^. Double-strand or loop suture procedures have made multi-strand repairs possible in recent years. With fewer suture bites and a doubling of the strands across the repair site, these sutures make the process easier. Its exceptional performance belies its exorbitant cost, which prevents hospitals from using it.

While performing the suturing techniques, it was noted that the two double-loop techniques-controlled gap formation better than the other two techniques. To highlight another point, apart from the four strands that cross within the tendon (core suture), by performing the two double-loop technique, extra strands crossed the outside of either side of the tendon repair site, which could act as a splint for the repaired tendon. In total, four strands crossed within the tendon repair site, and another two strands crossed either side of the repair site outside of the tendon substance. This possibly explains the added tensile strength that this repair produces, which makes it statistically significant compared to the other two well-established techniques.

Various preferences for flexor tendon repair techniques provide the patients with the best possible functional outcome^26^. Previously, a two-strand repair technique was considered strong. However, reports on rupture during early mobilisation protocols raise great concern^20^. Multiple-strand sutures improve the tendon tensile strength but inevitably increase the operational time, which poses tedious work for those discouraged from routine practice by hand surgeons.

When repairing a lacerated tendon, using more core sutures strengthens the repair and allows for a more vigorous rehabilitation with a lower risk of rupture. Ultimately, this will positively impact the reduction of adhesion formation potential. Unfortunately, because a bulkier repair creates more resistance, a repair with more core sutures will negatively affect tendon gliding. Internal knots are advantageous to facilitate tendon gliding. However, the possibility of ischemia and acellularity may ultimately result in partially or fully healed tendon repair and, in the end, rupture failure. Nowadays, a common technique in tendon restoration is using peripheral circumferential sutures, which ensure that the tendon's ends touch to increase strength and prevent rupture.

Identifying the ideal surgical approach to achieve successful results remains challenging for hand surgeons^15^. Several studies suggested primary flexor tendon repair. Strickland (1995) suggested that the suture materials should effortlessly pass through a tendon. The tendon ends should have a smooth junction and a well-secured suture knot, with a small gap at the wound healing site. This is crucial in order to avoid adhesion and resistance to tendon gliding. Conversely, vascularity should be minimally impacted by the repair construct^27,28^. Trail *et al*^23^ explain how the quantity of suture strands affects how resilient a flexor tendon repair and showed that, while they might raise tendon friction, 3-0 or 4-0 sutures significantly improve suture knot strength and reduce failure risks. Taras JS^29^ demonstrated that sutures significantly increase the strength of a repair with a bigger gauge. Furthermore, it has been shown by Soejima *et al*^30^ that the dorsal, as opposed to palmar, positioning of core sutures confers a notable biomechanical advantage. Nevertheless, even with the increased strength, the suture strands crossing the healing site will make the operation more laborious and probably cause nutritional compromise that will harm the tendon.

The two double-loop techniques also produce a minimal gap at the surgical site. The gap formed at the surgical site poses the weakest point of a tendon^9^, where it alters the biomechanical features of the tendon by promoting the formation of adhesions, which results in a reduced excursion. It was demonstrated by Diao *et al*^31^ that peripheral circumferential sutures are crucial following a core tendon repair. These sutures significantly reduce gapping between the tendon ends and a 10–50% increase in the strength of extensor tendon repair. Therefore, our study aims to prove the importance of the epitendinous suture upon flexor tendon repair. This is because it is believed that a repaired tendon's tensile strength increases dramatically with an increase in the number of core strands and epitendinous sutures^32^.

## Conclusion

This study postulates that the two double-loop techniques serve as an alternative suturing method for flexor tendon repair with a higher tensile load, are less technically demanding, and are more cost-effective. This method offers a noticeably strong tensile strength and is clinically acceptable, less complex, and comparatively straightforward. This would make it easier for medical professionals to treat many flexor tendon injuries surgically, quickly and effectively. However, we encourage a larger study involving questionnaire distribution among hand surgeons or multiple surgeons to determine the feasibility and suitability of performing this technique as their routine.
